# β-Caryophyllene: A Therapeutic Alternative for Intestinal Barrier Dysfunction Caused by Obesity

**DOI:** 10.3390/molecules27196156

**Published:** 2022-09-20

**Authors:** Uriel Ulises Rodríguez-Mejía, Juan Manuel Viveros-Paredes, Adelaida Sara Minia Zepeda-Morales, Lucrecia Carrera-Quintanar, José Sergio Zepeda-Nuño, Gilberto Velázquez-Juárez, Vidal Delgado-Rizo, Trinidad García-Iglesias, Luisa Guadalupe Camacho-Padilla, Elizabeth Varela-Navarro, Luis Alberto Anguiano-Sevilla, Esmeralda Marisol Franco-Torres, Rocio Ivette López-Roa

**Affiliations:** 1 Laboratorio de Investigación y Desarrollo Farmacéutico, Departamento de Farmacobiología, Centro Universitario de Ciencias Exactas e Ingenierías, Universidad de Guadalajara, Guadalajara 44430, Jalisco, Mexico; 2 Laboratorio de Análisis Quimícos Clínicos y Bacteriológicos Vinculación, Departamento de Farmacobiología, Centro Universitario de Ciencias Exactas e Ingenierías, Universidad de Guadalajara, Guadalajara 44430, Jalisco, Mexico; 3 Laboratorio de Ciencias de los Alimentos, Departamento de Reproducción Humana, Crecimiento y Desarrollo Infantil, Centro Universitario de Ciencias de la Salud, Universidad de Guadalajara, Guadalajara 44350, Jalisco, Mexico; 4 Departamento de Microbiología y Patología, Centro de Investigación y Diagnóstico de Patología, Centro Universitario de Ciencias de la Salud, Universidad de Guadalajara, Guadalajara 44350, Jalisco, Mexico; 5 Laboratorio de Bioquimíca Estructural, Departamento de Química, Universidad de Guadalajara, Centro Universitario de Ciencias Exactas e Ingenierías, Universidad de Guadalajara, Guadalajara 44430, Jalisco, Mexico; 6 Departamento de Fisiología, Centro de Investigación en Inmunología y Dermatología, Centro Universitario de Ciencias de la Salud, Universidad de Guadalajara, Guadalajara 44350, Jalisco, Mexico; 7 Departamento de Fisiología, Instituto de Investigación en Cáncer de la Infancia y Adolescencia, Centro Universitario de Ciencias de la Salud, Universidad de Guadalajara, Guadalajara 44350, Jalisco, Mexico; 8 Laboratorio de Biología Molecular, Genética y Proteómica, Instituto Transdiciplinar de Investigación y Servicios, Centro Universitario de Ciencias Exactas e Ingenierías, Universidad de Guadalajara, Guadalajara 45150, Jalisco, Mexico

**Keywords:** β-caryophyllene, dysbiosis, obesity

## Abstract

Obesity is an excessive accumulation of fat that exacerbates the metabolic and inflammatory processes. Studies associate these processes with conditions and dysregulation in the intestinal tract, increased concentrations of lipopolysaccharides (LPSs) in the blood, differences in the abundance of intestinal microbiota, and the production of secondary metabolites such as short-chain fatty acids. β-Caryophyllene (BCP) is a natural sesquiterpene with anti-inflammatory properties and with the potential purpose of fighting metabolic diseases. A diet-induced obesity model was performed in 16-week-old C57BL/6 mice administered with BCP [50 mg/kg]. A reduction in the expression of Claudin-1 was observed in the group with a high-fat diet (HFD), which was caused by the administration of BCP; besides BCP, the *phyla* *Akkermansia* and *Bacteroidetes* decreased between the groups with a standard diet (STD) vs. HFD. Nevertheless, the use of BCP in the STD increased the expression of these *phyla* with respect to fatty acids; a similar effect was observed, in the HFD group that had a decreasing concentration that was restored with the use of BCP. The levels of endotoxemia and serum leptin increased in the HFD group, while in the HFD + BCP group, similar values were found to those of the STD group, attributing the ability to reduce these in conditions of obesity.

## 1. Introduction

Obesity is a public-health concern worldwide that requires new approaches and a recognized international consensus in treating diseases leading to death [1]. The etiology of obesity is complex and includes biological and environmental factors that contribute to the consumption of a high-calorie diet and a reduction of physical activity. Indeed, in many high-income countries where sedentary lifestyles are becoming predominant, more than 60% of adults are considered overweight or obese [2]. Chronic obesity causes systemic inflammation and alters immunometabolic parameters that may result in the functional deterioration of the gastrointestinal barrier [3,4]. Dysfunction of the intestinal barrier integrity induced by toxins and pathogens is associated with a variety of gastrointestinal disorders and diseases, such as obesity [5]. In a murine model, the plasma LPS concentration increased 2–3 times during the administration of a 4-week high-fat diet (HFD), chronically termed “metabolic endotoxemia.” Moreover, endotoxemia increased or decreased during the feeding or fasting state, respectively, and increased the proportion of the LPS-expressing bacteria in the gut [6].

The gut microbiota plays an essential role in normal intestinal functions and the maintenance of the host’s health [7]. Consequently, an imbalance of the microbiota, or dysbiosis, can be the cause of the progression of various pathologies, such as infectious diseases, gastrointestinal cancers, inflammatory bowel disease, and even obesity and diabetes. A gut bacterial diversity is mainly affected by the diet. A high carbohydrate consumption leads to the proliferation of *firmicutes* and *proteobacteria*, while saturated fat and animal-protein consumption favor *Bacteroidetes* and actinobacteria. During their gastrointestinal passage, components of the diet, such as resistant starch and fiber, are substrates to fermentation products and the production of short-chain fatty acids (SCFAs), acetate, propionate, and butyrate [8]. The bacterial SCFAs provide an additional energy source and increase the synthesis of tight-junction proteins [5], and SCFAs stimulate the leptin production in adipocytes [9]. Studies indicate that circulating SCFAs, particularly acetate and propionate, can promote leptin secretion from adipocytes, providing an anorexigenic signal to the appetite-regulatory neurons in the hypothalamic arcuate nucleus [10].

Diet provides the nutritional supplies for life and growth, and some components exert beneficial effects when consumed regularly. These components are called “functional foods” or “nutraceuticals” [11]. β-Caryophyllene (BCP) is a nutraceutical and a natural bicyclic sesquiterpene that is the main constituent of the essential oils of numerous plants. It can be consumed in the diet in the form of oregano, cinnamon, and black pepper [3].

The aim of this study was to evaluate the modulatory effect of BCP on the integrity of the intestinal barrier, serum endotoxemia levels, the concentration of SCFAs, an abundance of bacterial *phyla* present in the colon, and serum leptin levels in a murine model of obesity induced by a hypercaloric diet.

## 2. Results

**Figure A1:**
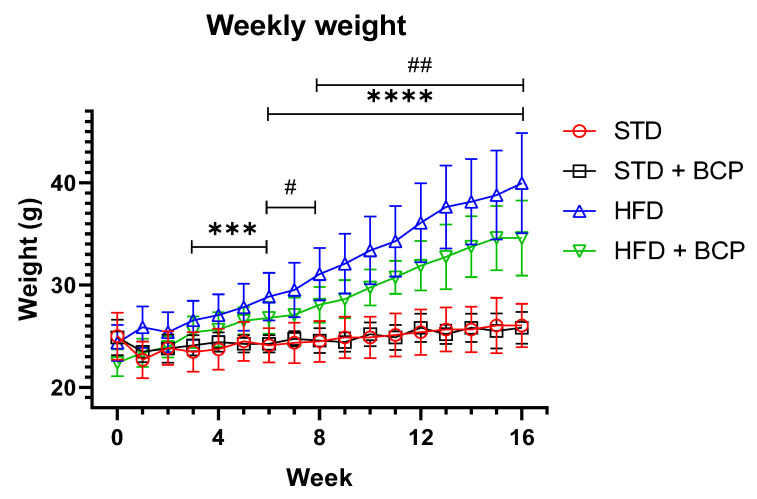
Effect of BCP on body weight gain of the study groups. * Significant difference between diet type, and # significant difference between HFD vs. HFD + BCP groups. Data are shown as mean ± standard deviation (SD), analyzed by one-way ANOVA/Tukey test. STD *n* = 11; STD + BCP *n* = 9; HFD *n* = 11; HFD + BCP *n* = 10. *** *p* < 0.001; **** *p* <0.0001, # *p* <0.05, and ## *p* < 0.01.

**Figure A2:**
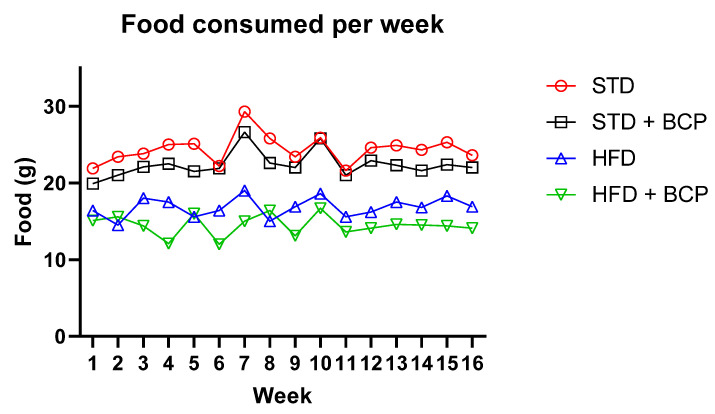
Food consumed per week of the study groups. This graph represents the average food consumption for each mouse in each of the study groups per week. STD *n* = 11; STD + BCP *n* = 9; HFD *n* = 11; HFD + BCP *n* = 10.

The diet-induced obesity model was properly implemented, replicating results already described with the BCP supplementation, such as a decrease in weight gain and immunometabolic parameters. Among the four experimental groups, we observed a significant difference between the two types of diet from week 3 (*p* < 0.001), and a difference between the groups on a hypercaloric food diet (HFD vs. HFD + BCP) at week 6 (*p* < 0.05) that increased and was maintained through to week 16 (*p* < 0.01) (Figure A1, Appendix B). The average food consumption in each week is shown in Figure A2.

### 2.1. β-Caryophyllene Increases the Number of Goblet Cells and the Thickness of Mucus in the Colon of Mice on a Standard Diet

**Figure 1:**
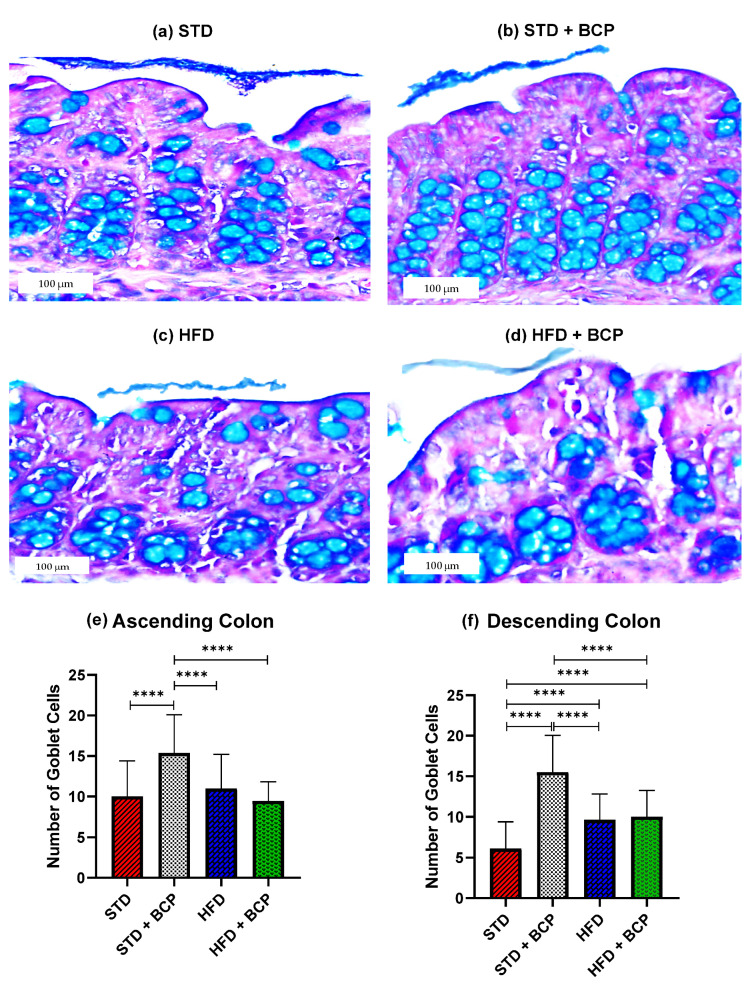
Goblet cells in the study groups. Histological images stained with Alcian blue; (**a**–**d**) Ascending colon in the study groups *n* = 3 for each group; (**e**) Number of goblet cells in the ascending colon, and (**f**) Number of goblet cells in the descending colon. Data are shown as mean ± SD, analyzed by one-way ANOVA/Tukey test., **** p < 0.0001.

**Figure A3:**
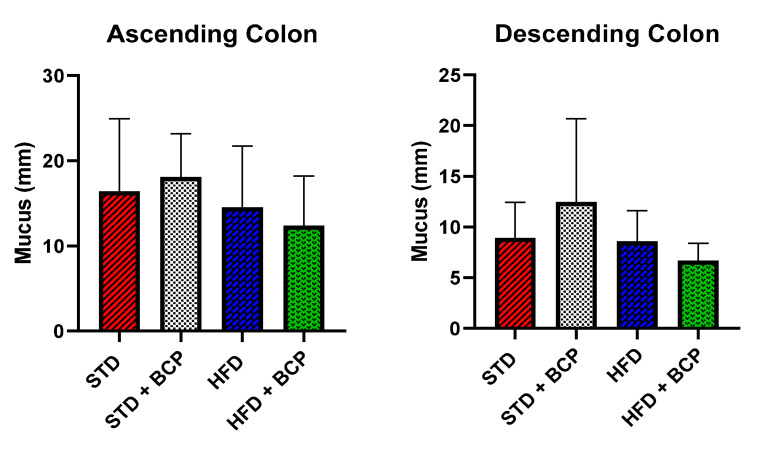
Effect of BCP on the mucus layer. Data are shown as mean ± SD, analyzed by one-way ANOVA/Tukey test, *n* = 3 for each group.

**Figure A4:**
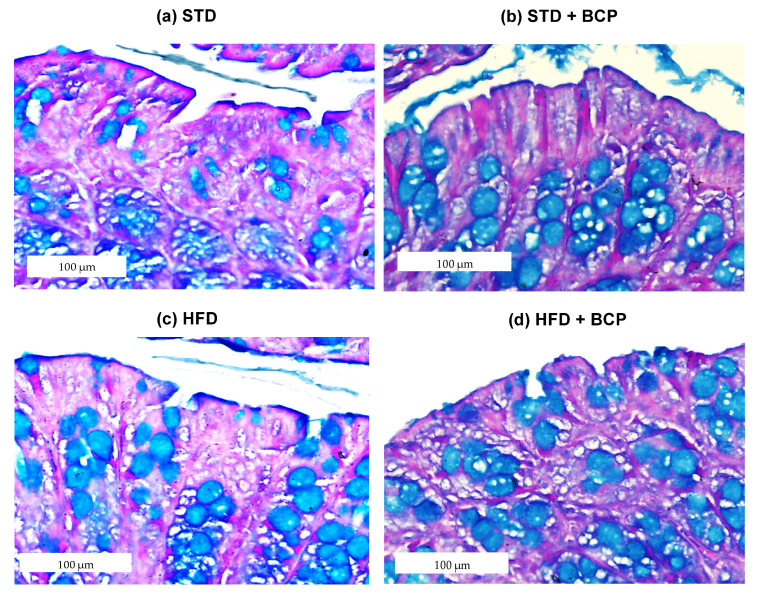
Goblet cells in the descending colon. Data are shown as mean ± SD, analyzed by one-way ANOVA/Tukey test, *n* = 3 for each group.

As shown in Figure 1, it was found that BCP can significantly increase the number of goblet cells per crypt in the ascending and descending colons under the conditions of a standard diet (STD). This finding is consistent with what was observed in the thickness of the mucus layer, showing a trend in the increased thickness in the STD + BCP group in both portions of the colon (Figure A3). Although the number of goblet cells in the STD group decreased in the descending colon, histologically larger clusters of cells were observed in comparison with the other groups, although this did not significantly impact the thickness of the secreted mucus (Figure A4).

### 2.2. β-Caryophyllene Restores the Claudin-1 Protein Expression in Mice with Diet-Induced Obesity

**Figure 2:**
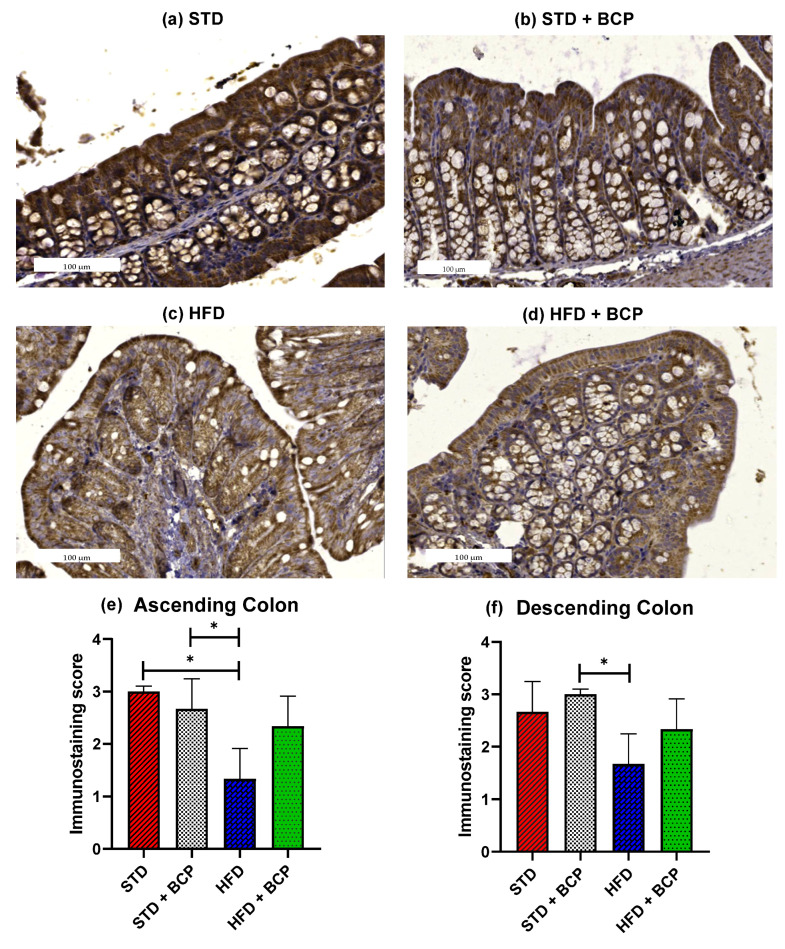
Claudin-1 protein expression in the colon. Histological images stained with immunohistochemistry; (**a**–**d**) Ascending colon in the study groups *n* = 3 for each group; (**e**) Immunostaining score in the ascending colon, and (**f**) Immunostaining score in the descending colon. Data are shown as mean ± SD, analyzed by one-way ANOVA/Tukey test. * p < 0.05.

**Figure A5:**
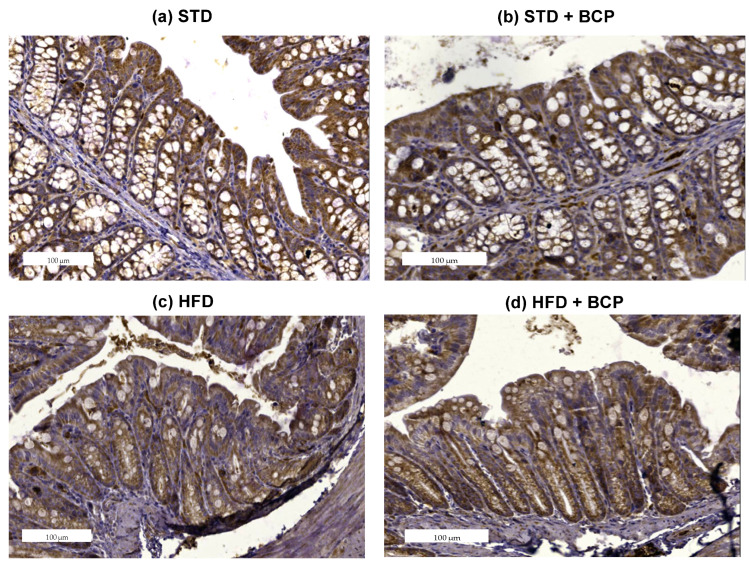
Claudin-1 protein expression in the descending colon. Histological images stained with immunohistochemistry (**a**–**d**). Descending colon in the study groups, *n* = 3 for each group.

The loss of intestinal barrier integrity is closely linked with the alterations in the abundance of tight junctions [12]. In the Claudin family, we found one of the proteins in which differences were reported and these differences were related to obesity [13]. To ascertain whether BCP has a beneficial effect on the integrity of the intestinal barrier, immunostaining of the colon was performed on both sections. A difference was observed in the expression of Claudin-1 in the ascending colon, between the STD vs. HFD groups (*p* < 0.05). The administration of BCP restored the protein expression of Claudin-1 in the HFD + BCP group, maintaining it slightly lower than that in the STD group (Figure 2). In relation to the descending colon, similar results were found in which the HFD + BCP group maintained a tendency to increase the expression of Claudin-1 compared with the HFD group (Figure A5).

### 2.3. β-Caryophyllene Decreases the Metabolic Endotoxemia Levels in Mice with Diet-Induced Obesity

**Figure 3:**
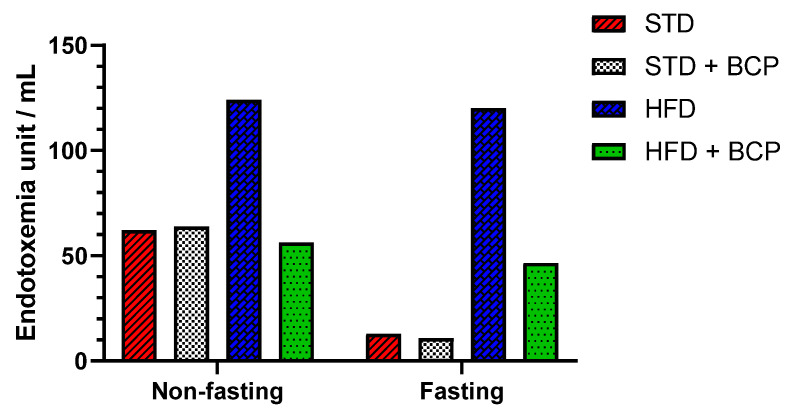
Serum LPS concentration in the study groups. Data is displayed in endotoxin units (EU)/mL.

The dysfunction of the intestinal barrier apparatus is associated with the increased intestinal permeability to LPSs, resulting in elevated systemic LPS levels that aggravate a low-grade inflammation [14]. In the quantification of serum LPS, two conditions were evaluated, “Fasting” and “Non-fasting,” before obtaining the sample (Table A1). A difference in the LPS concentration was observed in non-fasting mice among the STD, STD + BCP, and HFD + BCP groups vs. the HFD (Figure 3), where the HFD-diet group demonstrated twice as many endotoxin units (EU)/mL. Under the fasting condition, a 10-fold higher concentration was observed between the HFD group and the STD and STD + BCP groups, while in the HFD + BCP group, the values remained lower than those of the HFD group, but were constant with or without fasting.

### 2.4. β-Caryophyllene Selectively Modulates the Microbial Abundance in Feces

Obesity is associated with changes in the bacterial diversity where changes in the composition of microbiota are reported with a bacterial predominance [14,15], as well as the association of certain beneficial *phyla* in intestinal disorders, such as *Akkermansia muciniphila* [16] and *Fecalibacterium prausnitzzi* [17].

**Figure 4:**
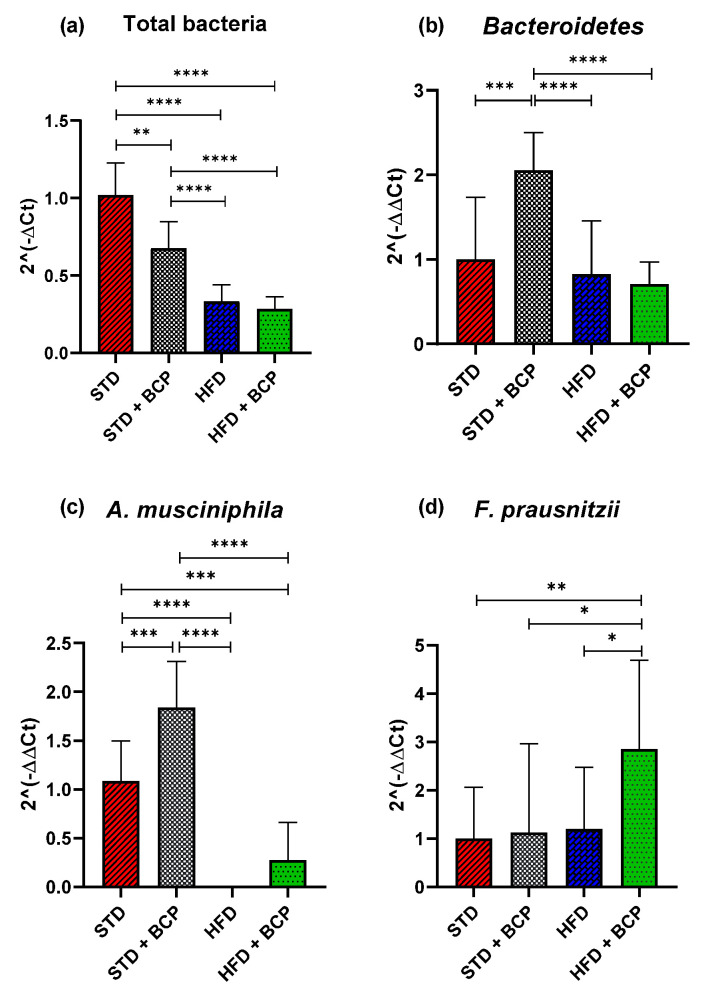
Relative expression of the bacterial *phyla* in the study groups. (**a**) Relative abundance of the total bacteria determined by the *16s* rDNA gene; (**b**) Relative abundance of *Bacteroidetes*; (**c**) Relative abundance of *A. muciniphila*, and (**d**) Relative abundance of *F. prausnitzii*. Data are shown as geometric mean ± geometric standard deviation (**a**,**b**,**d**); data are shown as mean ± SD (**c**), analyzed by one-way ANOVA/Tukey test. * *p* < 0.05, ** *p* < 0.01, *** *p* < 0.001, and **** *p* < 0.0001, (*n* = 9–11; STD = 9; STD + BCP = 9; HFD = 11; HFD + BCP = 10).

**Figure A6:**
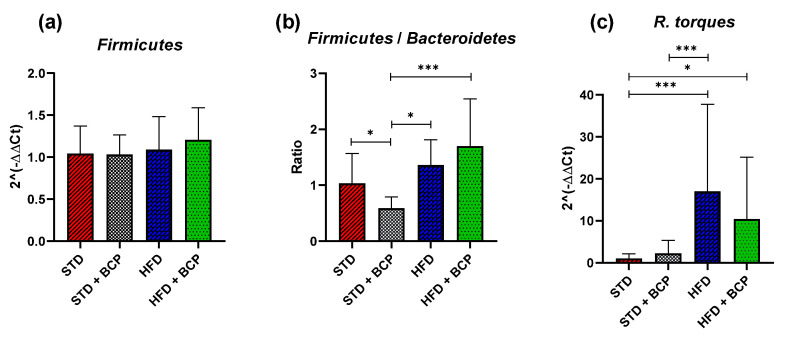
Relative expression of the bacterial *phyla* in the study groups. (**a**) Relative abundance of the *Firmicutes*; (**b**) Ratio of the *Firmicutes*/*Bacteroidetes*, and (**c**) and Relative abundance of the *R*. *torques*. Data are shown as geometric mean ± geometric standard deviation (**a**,**c**), data are shown as Mean ± SD, and (**b**), analyzed by one-way ANOVA/Tukey test. * *p* < 0.05; *** *p* < 0.001, (n = 9–11; STD = 9; STD + BCP = 9; HFD = 11; HFD + BCP = 10).

In the present study, the relative abundance of the total bacteria determined by the *16s* rDNA gene was considered, where a decrease in the number of total bacteria was observed in the two feeder groups with the HFD (*p* < 0.0001) and the STD + BCP group (*p* < 0.001), evidencing the selective antimicrobial activity of cannabinoid terpenes [18] (Figure 4a). Regarding the *phylum Bacteroidetes*, no significant difference was found among the STD, HFD, and HFD + BCP groups, but a two-fold increase in the gene expression was observed in the STD + BCP group (*p* < 0.001) (Figure 4b). The *phylum Firmicutes* did not significantly differ among the study groups (Figure A6). In the case of *A. muciniphila*, it was observed that the treatment with BCP increased its expression by about 1.8 times between the groups STD + BCP vs. STD (*p* < 0.001) and 0.3 times in the groups HFD + BCP vs. HFD, the latter not reaching any significance (Figure 4c). In the analysis of *F. prausnitzzi*, it was found that BCP increased its expression 2.8-fold between HFD + BCP vs the STD group (*p* < 0.01), thereby promoting the abundance of this *phylum* in high-fat diets (Figure 4d). For *Ruminococcus torques*, an increase was identified in the HFD group (*p* < 0.001), and the administration of BCP decreased the expression (*p* < 0.05) (Figure A6).

### 2.5. Influence of β-Caryophyllene on the Production of Metabolites of the Microbiota (Short Chain Fatty Acids) in the Ascending Colon and Feces

**Figure 5:**
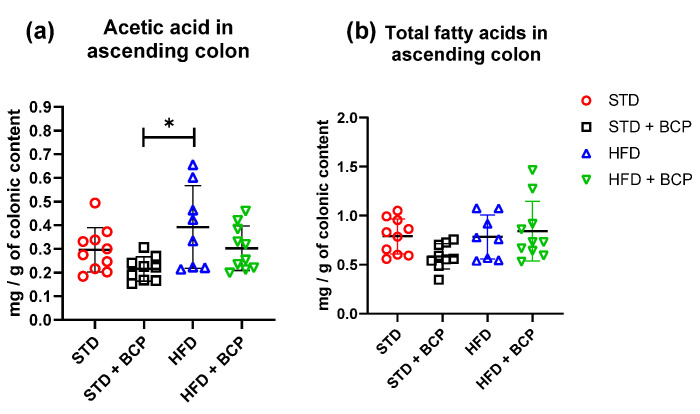
Concentration of SCFAs in the colonic content. (**a**) Concentration of acetic acid in the colonic content; and (**b**) Concentration of total fatty acids (acetic, propionic, and butyric acids) in the colonic content. Data are shown as mean ± SD, analyzed by one-way ANOVA/Tukey test. * *p* = 0.0109, (*n* = 8–10; STD = 10; STD + BCP = 9; HFD = 8; HFD + BCP = 10).

**Figure A7:**
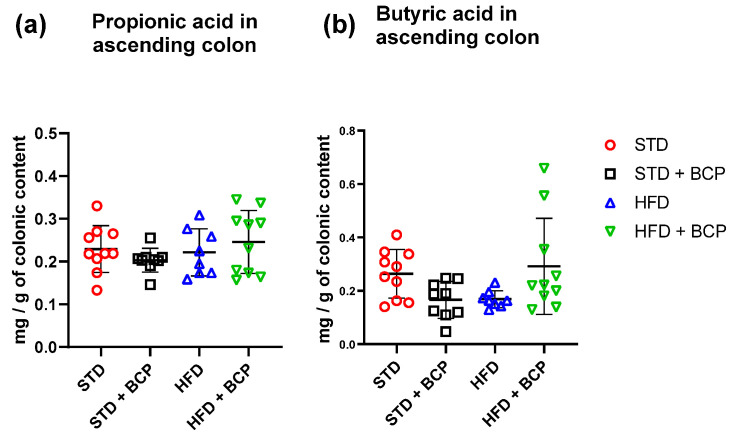
Short-chain fatty acid (propionic and butyric) concentration in the ascending colon. (**a**) Concentration of propionic acid in the ascending colon, and (**b**) Concentration of butyric acid in the ascending colon. Data are shown as mean ± SD, analyzed by one-way ANOVA/Tukey test. (*n* = 8–10; STD = 10; STD + BCP = 9; HFD = 8; HFD + BCP = 10).

In the quantification of SCFAs in the ascending colon (Table A2), a significant increase in acetic-acid concentrations was observed in the HFD group compared to the STD + BCP group (Figure 5). On the other hand, no significant difference was observed in the concentrations of propionic and butyric acid in the study groups (Figure A7).

**Figure 6:**
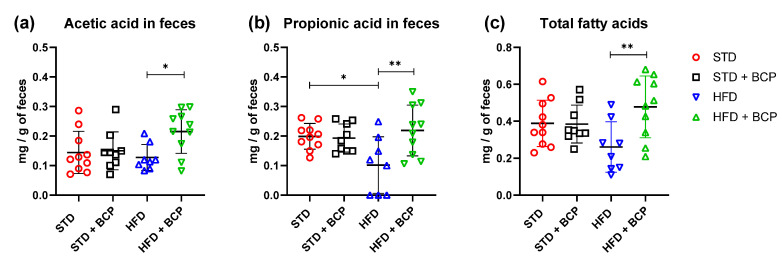
Concentration of SCFAs in feces. (**a**) Concentration of acetic acid in feces; (**b**) Concentration of propionic acid in feces, and (**c**) Concentration of total fatty acids (acetic, propionic, and butyric acids) in feces. Data are shown as mean ± SD, analyzed by one-way ANOVA/Tukey test. * *p* < 005 and ** *p* < 0. 001, (*n* = 8–10; STD = 10; STD + BCP = 9; HFD = 8; HFD + BCP = 10).

**Figure A8:**
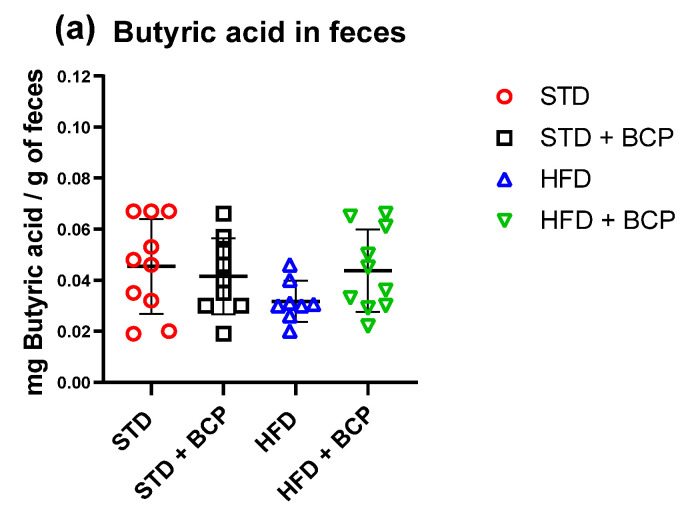
Short-chain fatty acid (butyric) concentration in feces. (**a**) Concentration of butyric acid in feces. Data are shown as mean ± SD, analyzed by one-way ANOVA/Tukey test. (*n* = 8–10; STD = 10; STD + BCP = 9; HFD = 8; HFD + BCP = 10).

In the determination of the short-chain fatty acids in feces (Table A3), a significant increase in acetic, propionic, and total fatty-acid concentrations was observed in the HFD + BCP vs. the HFD group, with results of *p* = 0.0378, *p* = 0.0072, and *p* = 0.0099, respectively (Figure 6). There was a re-establishment of the concentration of propionic acid in the HFD + BCP group, equating it to the concentrations of the groups with the STD. Regarding the concentration of butyric acid, no significant difference was found among the study groups, but a tendency was observed in terms of the increase of its concentration (Figure A8).

### 2.6. β-Caryophyllene Administration Decreases the Serum Leptin Levels in Mice

The leptin expression and signaling are regulated by many factors, including hormones and dietary components. Although adiposity is the primary determinant of leptin levels, the gut microbiota can regulate leptin synthesis and/or signaling. SCFAs are metabolites of colonic bacteria; the G protein-coupled receptors (FFAR2 and FFAR3) are the main receptors for SCFAs. The circulating SCFAs have been reported to interact with different organs and tissues. In particular, they have been shown to play an important role in the adipocyte function and metabolism, and the stimulation of leptin secretion.

**Figure 7:**
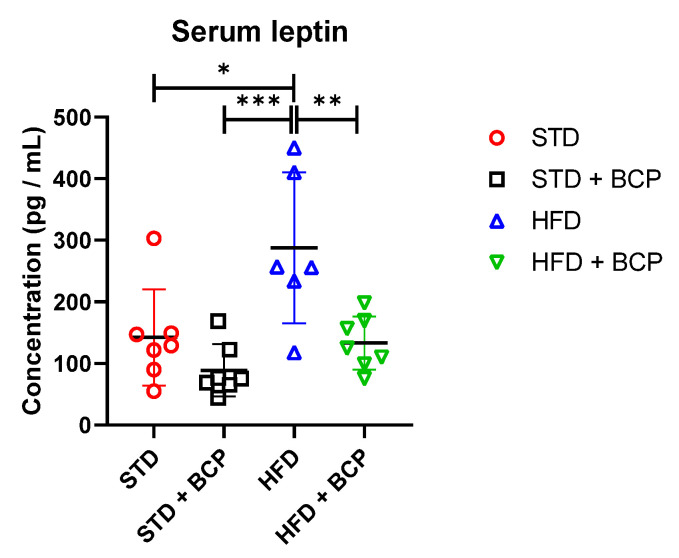
Concentration of serum leptin in the study groups. Data are shown as mean ± standard deviation (SD), analyzed by one-way ANOVA/Tukey test. * *p* = 0.0115; ** *p* = 0.0071, and *** *p* = 0.0006 (*n* = 6–7; STD = 7; STD + BCP = 7; HFD = 6; HFD + BCP = 7).

BCP decreases the leptin concentration regardless of the type of diet consumed compared with its untreated counterpart group (Table A4). It was observed that the administration of BCP significantly reduced the leptin concentration in the HDF + BCP group (Figure 7).

## 3. Discussion

In the present study, the administration of BCP at a dose of 50 mg/kg had an effect on body weight gain associated with diet-induced obesity (DIO), in agreement with that reported by Franco-Arroyo et al. (2022) [3] in a murine model of C57BL/6 mice with a hypercaloric diet, where a difference in the weight gain was found between HFD vs. HFD + BCP mice from week 8, though we observed a difference from week 6.

Regarding this result, Verty et al. 2015 [19] reported that mice of the C57BL/6 strain that were fed an HFD diet ad libitum, decreased their body weight after 21 days of the intraperitoneal administration of JWH-015 (10 mg/kg), which is a CB2 receptor agonist. The main findings of this study demonstrate, to our knowledge for the first time, that the chronic administration of a CB2 receptor agonist produces a reduction in body weight in DIO mice despite a transient reduction in food intake.

Cannabinoids exert numerous effects on the gastrointestinal tract ranging from the modulation of food intake, visceral sensation, inflammation, and intestinal permeability [20]. This permeability is mainly associated with mucus secretion and tight-junction proteins, with findings reported in the mucus thickness and the Claudin-1 protein expression in the colon, such as those of Gulhane et al., where the authors mentioned that there is no difference in the amount of mucus secreted. Nonetheless, these authors reported a decrease in the differentiation of goblet cells, the gene expression of *Muc2*, and the loss of the Claudin-1 protein expression in a murine model of DIO mice [21]. In addition, no difference in the thickness of the mucus in an ob/ob obesity model was ascribed by Schroeder et al. [22].

These results are in agreement with those obtained in the present study, in that we did not find any significant differences in the thickness of the mucus layer among the groups, and a decrease in the protein expression of Claudin-1, observed in the group with induced obesity, restored the values to those comparable to the standard in the HFD + BCP group. In the goblet-cell-count results, Lin et al. showed that the use of HU-210, a non-selective agonist of CB1 and CB2 receptors, prevented the decrease in goblet cells in the colon caused by colitis induced by dextran sulfate sodium (DSS) [23].

The use of BCP increases the abundance of goblet cells in animals with a STD. To date, there have been, to our knowledge, no studies on the uses and effects of CB2 receptor agonist phytocannabinoids regarding the abundance and differentiation of the intestinal cells in models of obesity. Notwithstanding this lacuna, such a study with the results of a study reported by Lin et al., opens the question concerning the effects and actions of the activation of endocannabinoid receptors and their influence on the modulation of the diversity and cellular composition of the intestinal epithelium. This study will serve as a basis and reference for future research in this matter.

The intestinal epithelium is an effective barrier that prevents the absorption of LPSs. Structural changes in the intestinal epithelium in response to alterations in the diet allow LPSs to enter the bloodstream, thus increasing the plasma levels of the LPSs, that is referred to as metabolic endotoxemia [24]. Research carried out by Gulhane [21], Muccioli [25], and Cani [5] demonstrate that the increase in serum LPS, is attributed to the type of diet consumed by murine and human models and to the fasting condition. Specifically, Muccioli demonstrated that the intestinal permeability is associated with the activation of CB1 receptors through stimulation with HU-210 and the selective blockers for CB1 and CB2 receptors. Cani confirmed these results in his review, distinguishing between ‘gate keepers’ or ‘gate openers’ in terms of the agonists and the abundance of CB2 and CB1 receptors in the intestine, which are associated with serum LPS levels [26].

Our results are in agreement with those described by these researchers, combining the information in which the high-fat diet increases the level of serum LPS, and coinciding with what was reported by Cani in 2007 [6], where the fasting or non-fasting condition affects the levels of endotoxemia, and as observed, the levels increase under non-fasting conditions. As discussed in our study, we reported a fivefold increase of in the LPS concentration between both STD groups in the two conditions, while the hypercaloric-diet group maintained the same levels with or without fasting, thereby demonstrating a difference of 10 times more LPSs between the STD and HFD groups under fasting conditions.

Different types of diets can modulate the intestinal microbiota by developing specific bacterial profiles for various disorders and pathologies [8]. Research studies conducted by Ley [27], one of the first authors to report on a condition of dysbiosis associated with metabolic disorders, mentions a change in the proportions of the *phyla*, increasing the amount of *Firmicutes* and decreasing that of *Bacteroidetes*. Later studies, such as those by Hilderbrandt [28] and de Wit [29], are in agreement with what was reported by Ley. In our case, we did not observe a significant difference in the F/B ratio, but instead, an increasing trend in the HFD vs. STD group. Authors such as Bisanz [30] reported that not all studies maintain such conclusive results with respect to the F/B ratio, but a correlation is observed between the increase in the ratio and consumption of the HFD. We are also in agreement with that observed by Magne et al. [31], in which patients with obesity did exhibit less bacterial diversity than lean subjects, but suggests the existence of other changes at the genus or species levels.

Regarding the results obtained in the number of total bacteria through the analysis of the *16s* gene, we observed a decrease in the groups administered with BCP. In this regard, we are in agreement with the findings reported by Dahman [32] and Yoo [33], where the authors demonstrated the antimicrobial and antifungal activity of BCP and a decrease in bacterial diversity with a more pronounced activity against the gram-positive than against the gram-negative bacteria. We observed this microbial selective modulation in the amount of *Bacteroidetes* in the present study where, despite the bacterial decrease in the groups with the BCP treatment in conjunction with a standard diet, the expression of *Bacteroidetes* increased significantly, evidencing a diet-dependent selective mechanism by the BCP.

Moreover, we observed similar responses in the *A. muciniphila* and *F. prausnitzii* *phyla*, in that the groups administered with BCP demonstrated an increase in the abundance of *Akkermansia.* Specifically, the HFD + BCP group showed a considerable increase in *Faecalibacterium*. Our results are similar to those reported by Wang et al. and López-Silés et al., in which the authors specified that the metabolites released by *A. muciniphila* promoted the proliferation of *F. prausnitzii*, which converted acetate and lactate into butyrate, also demonstrating that *A. muciniphila* and *F. prausnitzii* co-exist in the mucosa and decrease the symptoms in inflammatory bowel disease [34,35].

Liang et al. render explicit the relation between the butyrate levels and the increase in mucin production and the number of goblet cells in the colonic crypt [36]. Furthermore, we considered what was reported by Crouch et al., that the main mechanism for obtaining energy from *Akkermansia* is through the degradation of mucin, [37]. The latter is due to the close relationship between *Akkermansia* and *Faecalibacterium*. In addition, there is homeostatic mechanism in which concentrations of butyric acid promote a greater number of goblet cells with the ability to secrete richer mucus in mucin, thus creating feedback with the pathway dependent on a diet with the presence of fibers, which is lost when modified by a high-fat diet.

The fermentation metabolites (acetic, propionic, and butyric acid) from dietary fibers, is associated with various benefits by stimulating multiple hormonal and neural pathways [38,39], appetite control, energy homeostasis [40], reduction of body weight [39,41], among others. To date, there are several studies in which the results are contrasting, but in the majority of studies, a decrease in SCFAs is observed in the DIO groups [41,42,43]. In our study, we did not find significant differences in the ascending colon but, in feces, we did observe a decrease in fatty acids in the HFD group. Moreover, the administration of BCP increased the concentration of acetic acid and propionic acid, even above that of the STD groups.

Research such as that carried out by Chambers et al. [10] mentions a possible role of fatty-acid receptors in the regulation of food intake due to the production of anorexigenic hormones, such as peptide tyrosine-tyrosine (PYY), glucagon-like peptide (GLP)-1 by the colonic L cells, and leptin by adipocytes [44]. Although adiposity is the main determinant of leptin levels, the gut microbiota can also regulate leptin synthesis and/or signaling. SCFAs, through their G protein-coupled receptors (FFAR2 and FFAR3), and these have been shown to play an important role in adipocyte function and metabolism and the stimulation of leptin secretion.

Xiong et al. reported that the FFAR3 expressed in adipocytes via SCFAs can stimulate the leptin expression in a mouse adipocyte cell line and in mouse adipose tissue in primary culture. Studies indicate that circulating SCFAs, particularly acetate and propionate, can promote leptin secretion from adipocytes through the activation of the FFAR2, thus providing an anorectic signal [44]. Jiao et al. reported that the administration of sodium acetate and butyrate in HFD-fed animals significantly increased the leptin levels compared with the control group. The administration of these SCFAs resulted in a higher satiety and a lower energy intake, which is considered a good strategy to combat obesity [45]. Den Besten et al. reported, in a murine model of obesity-induced mice through an HFD diet, that the administration of SCFAs activated the expression of leptin through the SCFA receptor GPR43. However, these authors found reduced serum levels of the satiety factor leptin in SCFA-treated animals. This can be explained by the general decrease in white adipose-tissue mass. The same observation was previously reported in mice overexpressing GPR43. Despite their lower leptin levels, the food intake of the SCFA-treated animals did not increase. This may be due to other satiety factors that were not further investigated [41].

The results are in agreement with what has been described, because the levels above those of the standard group were obtained in the serum leptin and in acetic acid from the ascending colon. Thus, it can be thought that the concentrations of fatty acids in portions closer to the cecum entertain the possibility of absorbing these metabolites, thus increasing the concentration of the serum leptin. The results are similar to those reported by Pathak et al., who evaluated the effect of BCP on the hyperreactivity of the airways associated with obesity. These authors reported that animals fed an HFD diet had significantly increased leptin levels compared with their counterparts that were fed a standard diet, while the BCP administration significantly reduced the leptin concentrations, thereby improving hyperleptinemia [46].

## 4. Materials and Methods

An experimental study was conducted by means of a murine model with mice of the C57BL/6 strain, developed at the University Center for Exact Sciences and Engineering (CUCEI), University of Guadalajara, Mexico, in the Animal Room, the Experimentation Room, and in the facilities pertaining to the Research Laboratory and Pharmaceutical Development (LIDF) of the Department of Pharmacobiology.

### 4.1. β-Caryophyllene

The β-Caryophyllene (BCP) used in this protocol was kindly donated and characterized by Prof.Jürg Gertsch of the Institute of Biochemistry and Molecular Medicine at the University of Bern, Switzerland, as being 98% pure. The essential oil was stored at 4 °C and protected from light.

### 4.2. Diet

The standard diet consisted of 4.07 Kcal/g: 18.3% calories from protein, 59.6% calories from carbohydrates, and 22.1% calories from fat (Basal Diet 5755, Labdiet, St. Louis, MO, USA). The composition of the diet rich in saturated fat was 5.1 Kcal/g: 18.1% calories from protein; 20.3% calories from carbohydrates, and 61.6% calories from fat (DIO Rodent Purified Diet w/60% Energy from Fat-Blue 58Y1 (Labdiet, St. Louis, MO, USA).

### 4.3. Animals

Forty-five 8-week-old male C57BL/6 mice weighing between 22 and 26 g were obtained from the Universidad Autónoma Metropolitana (UAM, Xochimilco, Mexico). The animals were housed at a room temperature of 25 °C on a 12 h daylight cycle and were allowed food and water ad libitum, except during the tests that required fasting. On behalf of the Institutional Committee for Care and Use of Laboratory Animals (CICUAL) at the University Center of Exact Sciences and Engineering (CUCEI) of the University of Guadalajara, this protocol was approved with the registration number CUCEI/CINV/CICUAL-01/2022 (April 2022). All animal procedures were conducted according to the production, care, and use of laboratory animals established in the Mexican Official Standard (NOM-062-ZOO-1999).

### 4.4. Experimental Design

Following one week of acclimatization with ad libitum access to water and a standard diet, the mice were randomly separated into four groups: the standard diet (STD) (*n* = 11); the standard diet plus β-Caryophyllene (STD + BCP) (*n* = 9); the high-fat diet (HFD) (*n* = 11), and the high-fat diet plus β-Caryophyllene (HFD + BCP) (*n* = 10). The BCP groups received treatment with a dose of β-Caryophyllene (50 mg/kg of mouse weight). The treatment was administered daily by oral gavage technique for 112 days (16 weeks); for its administration, it was dissolved in the vehicle solution, which consisted of a 4% saline solution with Tween 80 (Sigma Aldrich, St. Louis, MO, USA). Finally, the animals were sacrificed by decapitation for the extraction of whole blood and for tissue dissection.

### 4.5. Goblet Cell Count

The paraffin blocks of the descending colon tissue were cut into 5-μm sections. For each sample, one slide containing five tissue sections was prepared. The sections were stained with Alcian blue (HYCEL, Zapopan, Mexico) and counterstained with hematoxylin to visualize the goblet cells. The number of goblet cells per sample was obtained from three randomly selected sections of the colonic epithelium. From each selected section, 10 crypts were randomly chosen (30 crypts in total per sample). The quantity of goblet cells was measured using APERIO Image Scope software (https://www.leicabiosystems.com/es/patologia-digital/gestion/aperio-imagescope/) (accessed on: 24 March 2022) by LEICA at a 20-X magnification (100 μm). Therefore, the number of goblet cells was expressed as the “number of goblet cells per crypt per 100 µm” [47].

### 4.6. Immunohistochemistry

The Claudin-1 protein expression was determined in the ascending and descending colon tissue. The resected tissue was fixed immediately with 10% formaldehyde in 1X phosphate buffer saline (PBS), processed, and embedded in paraffin. The paraffin-embedded samples were cut and the sections (3-µm) were treated with the Novolink^®^ polymer detection system (Leica Biosystems, Buffalo Grove, IL, USA), according to the manufacturer’s instructions. The antigen retrieval was performed with an EDTA buffer (1 mM EDTA, 0.05% Tween 20, and pH 9.0) for 40 min.

The sections were incubated overnight at 4 °C with a mouse monoclonal primary antibody (sc-166338; Santa Cruz Biotechnology, Danvers, MA, USA) at a 1:250 dilution. The adipose tissue was used as the negative control. Finally, the chromogen working solution and hematoxylin were employed to perform the detection and the counterstain, respectively. The Claudin-1 expression was documented using Aperio LV1 IVD equipment and Aperio image scope software (Leica Biosystems, Buffalo Grove, IL, USA). The staining intensity was scored as follows: 0 = negative; 1 = weak; 2 = moderate, and 3 = strong.

### 4.7. LPS Quantification

The LPS quantification was performed using the Limulus Amebocyte Lysate (LAL) method with the Endosafe PTS^®^ cartridge with a quantification range of 0.05–5 endotoxin units (EU)/mL of the Charles River brand (Cat. CRLPTS2005F). Each cartridge provides duplicate sample analysis and reports the result in EU/mL and % C.V.

A mixture of the serum was made from three mice of the same group with a similar weight and closest to the group average. The processing was carried out according to the manufacturer’s specifications.

### 4.8. Microbial Abundance (qPCR)

The genomic DNA was extracted from 20–30 mg of fecal samples using the Quick-DNA Fungal/Bacterial Miniprep Kit (Cat. D600; Zymo Research, Irvine, CA, USA), according to the manufacturer’s instructions. The DNA concentrations were measured by Nanodrop (Thermo Fisher Scientific, Waltham, MA, USA).

For the gene expression, the green-based SYBR (Cat. 172-5270; Bio-Rad, Santa Rosa, CA, USA), a real-time quantitative PCR (qPCR) assay was performed on a Rotor-Gene Q (Qiagen, Hilden, Germany), and the relative-expression ratios were determined by Rotor-Gene Q version 2.3.4 software (17 January 2022).

The primers were designed in line with the primer-BLAST tool and are listed in Table 1; *16s* rDNA was used as the reference gene. The procedure was carried out with a 10-µL reaction mix containing 5 µL of the SYBR Green enzyme, 0.4 µL of each forward and reverse primer, 2.2 µL of water, and 2 µL of DNA under the following conditions: denaturation at 95 °C for 30 s, with between 25 and 0 cycles at 95 °C for 15 s, at the hybridization temperature (varying for each gene) for 15 s, an extension of 72 °C for 30 s solely for products of more than 120 bp, and the melt ramp from 45–95 °C.

### 4.9. Short-Chain Fatty Acids (Gas Chromatography)

The samples were obtained at the end of the experiment (day 112) for the quantification of the short-chain fatty acids. These were stored at −80 °C until the day-of-processing. For the SCFA quantification, 20–30 mg of feces and 200 µL of water were added to each tube and this was stirred until homogenized. Once completed, this was aggregated with 40 μL HCl 0.1 M, 20 mg citric acid, and 40 mg of NaCl. Then, this was added to the homogenate: 200 μL of the solution prepared with N-butanol, tetrahydrofuran, and acetonitrile at a 50:30:20 ratio. All of the components were mixed for 1 min. The supernatant was obtained by centrifugation at 13,000× *g* for 10 min [48]

The supernatant was filtered with a Whatman^®^ GD/X syringe filter with 0.22 μm of PVDF (MERCK, Billerica, MA, USA). Once completed, the supernatant was analyzed in a Shimadzu GC 2010 plus gas chromatograph with a flame ionization detector (FID) (Shimadzu Scientific Instruments, Kyoto, Japan). In order to perform the analysis, a Mega-Acid^®^ high-polarity stationary-phase column (MEGA, Legnano, MI, Italy) was used.

The data were analyzed with LabSolutions, Chromatography Data System software (Shimadzu Scientific Instruments, Kyoto, Japan). In addition, the quantifications of each acid (acetic acid, butyric acid, and propionic acid) were analyzed with a standard 8-point curve (400, 200, 100, 50, 25, 12.500, 6.250, and 3.125 ppm). The concentration of SCFAs in each sample was determined by the interpolation of the data with the standard curve.

### 4.10. Serum Leptin Concentration (ELISA)

Serum leptin levels were quantified using the ELISA technique with the Mouse Leptin ELISA Kit^®^ (Cat. ELM-Leptin; RayBio, Gwinnett, GA, USA), according to the manufacturer’s specifications.

### 4.11. Statistical Analysis

A one-way ANOVA, Student *t* test, or Kruskal–Wallis test was performed, corresponding to the behavior of the data. *p* < 0.05 was established as significant and it was determined with the Tukey–Kramer or Dunn multiple statistics test using GraphPad Prism^®^ 6 software (version 19.0; IBM, Inc., Chicago, IL, USA).

## 5. Conclusions

The administration of BCP at a dose of 50 mg/kg prevents or reduces alterations in the intestinal barrier in the colon caused by obesity in the expression of Claudin-1, as well as a decrease in serum levels of endotoxemia and leptin, and an activity in bacterial dysbiosis, in which it acts as a selective regulator of the diet-dependent *phyla*, in addition to a possible stimulating effect on the differentiation of goblet cells.

## Figures and Tables

**Table 1:** Primers used for qPCR.

Gene	Forward	Reverse	Product	Temperature
16srDNA	AGTTTGATCCTGGCTCAG	GWATTACCGCGGCKGCTG	516	62 °C
*Bacteroidetes*	GGARCATGTGGTTTAATTCGATGAT	AGCTGACGACAACCATGCAG	126	63 °C
*Firmicutes*	GGAGYATGTGGTTTAATTCGAAGCA	AGCTGACGACAACCATGCAC	129	60 °C
*A. muciniphila*	CAGCACGTGAAGGTGGGGAC	CCTTGCGGTTGGCTTCAGAT	329	60 °C
*F. prausnitzii*	GATGGCCTCGCGTCCGATTAG	CCGAAGACCTTCTTCCTC	129	57 °C
*R. torques*	AATCTTCGGAGGAAGAGGACA	ACACTACACCATGCGGTCCT	137	56 °C

## Data Availability

Not applicable.

## Appendix A

### Appendix A.1. Calculation of the Sample Size

In order to determine the number of mice per group to be used, the results obtained during the first pilot will be considered. The variable to consider will be the body weight of the mice on a hypercaloric diet. The calculation will be made based on the formula for estimating the mean:

$$n=\frac{Z^{2}\sigma^{2}}{\delta^{2}}$$

*n* = sample size
Z = confidence level considering the *p* to be used = 1.96
σ = variation obtained in the measured parameter = 5.6
δ = desired variation of the parameter = 3

### Appendix A.2. LPS Quantification

For the quantification of the LPSs, it is essential to carry out an adequate sample collection due to the high probability of environmental contamination and the instability of the analyte. All of the material used was previously sterilized with moist heat and later with dry heat at a temperature of 120 °C for 8 h. In our case, whole blood was collected in glass chromatographic vials using an intraocular (i.o.) puncture with a borosilicate capillary without heparin. Once the sample was obtained, it was maintained at room temperature for 10 min and centrifuged at 2000 rpm for 10 min. The serum was separated and stored again in glass chromatographic vials at −80 °C until analysis. The analysis was performed in less than 7 days after obtaining the sample.

### Appendix A.3. Short-Chain Fatty Acids (Gas Chromatography)

The samples were taken from each of the mice and the anus and perianal areas were rendered aseptic with a Mycrodacyn^®^ solution (SAFER, CDMX, MX). Following stimulation with an abdominal massage, the fecal matter was placed directly into a sterile 2-mL microtube and introduced into the liquid nitrogen during the collection of the other samples.

In order to perform the analysis, a high-polarity stationary-phase Mega-Acid^®^ column (MEGA, Legnano, MI, Italy) was used, with the following characteristics: 30 m × 0.25 mm of the internal diameter (id) and a thickness of 0.40 μm. The conditions for the analysis were as follows: injection of 3 μL; ratio split 1:25; oven temperature 250 °C; detector temperature 250 °C; gas flow N_2_ 30 mL/min, H_2_ 40 mL/min, and air 400 mL/min.

## Appendix B

### Appendix B.1. Effect of β-Caryophyllene on Body Weight Gain

In the weight gain, a difference was observed from week 3 between the STD vs. HFD diet groups, which was increased, and a difference between the HFD vs. HFD + BCP diet groups, from week 6 (Figure A1). Regarding the food consumption during the study period, it was observed that the groups with a hypercaloric diet consumed less food compared with the standard diet (Figure A2).

### Appendix B.2. Effect of β-Caryophyllene on the Goblet Cell Numbers and the Mucus Layer

The results obtained by analyzing the thickness of the mucus in both portions of the colon do not show a significant difference among the study groups, but there was a trend consistent with the increase in goblet cells in the STD + BCP group.

### Appendix B.4. Effect of β-Caryophyllene on the Serum LPS Concentration (Endotoxemia)

**Table A1:** Serum LPS concentration.

			Sample		Control/Spike		
	ID	Dilution	EU/mL	% C.V.	EU/mL	% C.V.	% Recovery
Non-Fasting	STD	1:100	62.1	1.2	1.73	2.5	199
	STD + BCP	1:100	63.8	11.6	1.59	7.4	183
	HFD	1:1000	124	0.2	1.61	6.2	185
	HDF + BCP	1:100	56.2	2.4	0.678	5.6	78
Fasting	STD	1:100	12.8	2.2	0.690	2.7	79
	STD + BCP	1:100	10.8	16.2	0.867	3.3	100
	HFD	1:1000	120	0.7	1.19	10.4	137
	HDF + BCP	1:100	46.5	18	1.46	5.3	168

Table A1. The table presents the concentration of LPSs in serum and the coefficient of variation of each measurement made. The measurement is considered adequately analyzed if it complies with % C.V. < 25% and recovery % between 50–200%.

### Appendix B.5. β-Caryophyllene on the Microbial Abundance in Feces

The relative expression of the bacterial *phyla*- Figure A6a shows the results obtained from the gene expression of *Firmicutes*, which does not present significant differences, but instead a slight increase in the HFD + BCP group. Figure A6b presents the F/B ratio, a metabolic indicator still under discussion regarding its use; the increase in the index of this indicator is associated with metabolic syndromes [31]. Among our results, we obtained an increase in the HFD vs. STD group, but without a statistically significant difference. In the STD + BCP group, a reduction of one half in the ratio is observed, this is due to the increase found in the *phylum Bacteroidetes* (Figure 4). For the species of *Ruminococcus torques*, a 17-fold increase in the expression was observed between the HFD vs. STD groups, and there was a slight decrease reaching up to 10-fold between the HFD + BCP vs. STD groups.

### Appendix B.6. Concentration of the Short Chain Fatty Acids in the Colonic Content and Feces

**Table A2:** Concentration of SCFA in the ascending colon. The table presents the concentrations of acetic, propionic, butyric, and total fatty acids.

	STD	STD + BCP	HFD	HFD+ BCP
Acetic acid	0.295 ± 0.093	0.215 ± 0.051 *	0.392 ± 0174 *	0.302 ± 0.093
Propionic acid	0.229 ± 0.054	0.203 ± 0.027	0.221 ± 0.055	0.245 ± 0.073
Butyric acid	0.264 ± 0.090	0.166 ± 0.061	0.169 ± 0.031	0.291 ± 0.179
Total fatty acids	0.788 ± 0.179	0.584 ± 0.129	0.782 ± 0.224	0.840 ± 0.305

Units: mg of fatty acid/g of colonic content. mean ± SD. ** p* = 0.0109.

**Table A3:** Concentration of SCFA in feces. The table presents the concentration of acetic, propionic, butyric, and total fatty acids in feces.

	STD	STD + BCP	HFD	HFD+ BCP
Acetic acid	0.144 ± 0.713	0.150 ± 0.064	0.127 ± 0.044 *	0.215 ± 0.073 *
Propionic acid	0.199 ± 0.043 #	0.193 ± 0.047	0.101 ± 0.095 #**	0.219 ± 0.085 **
Butyric acid	0.045 ± 0.018	0.041 ± 0.014	0.031 ± 0.008	0.043 ± 0.016
Total fatty acids	0.388 ± 0.125	0.385 ± 0.102	0.261 ± 0.136 **	0.478 ± 0.166 **

Units: mg of fatty acid/g of feces. mean ± SD. # *p* = 0.0378, * *p* < 0.05, and ** *p* < 0.001.

### Appendix B.7. Concentration of Serum Leptin Levels in Mice

**Table A4:** Concentration of serum leptin. The table presents the concentration of serum leptin.

	STD	STD + BCP	HFD	HFD+ BCP
**Leptin**	142.2 ± 78.30	89.6 ± 42.48	287.5 ± 122.5	133.4 ± 43.14

Units: pg/mL. Mean ± SD.
